# Tyrosine supplementation is ineffective in facilitating soccer players’ physical and cognitive performance during high-intensity intermittent exercise in hot conditions

**DOI:** 10.1371/journal.pone.0317486

**Published:** 2025-01-16

**Authors:** Kate J. Donnan, Emily L. Williams, Nicholas Stanger

**Affiliations:** 1 Department of Sport, Health and Exercise Science, University of Hull, Hull, United Kingdom; 2 Carnegie School of Sport, Leeds Beckett University, Leeds, United Kingdom; Tokat Gaziosmanpasa University Tasliciftlik Campus: Tokat Gaziosmanpasa Universitesi, TÜRKIYE

## Abstract

Tyrosine has been proposed to potentially provide ergogenic benefits to cognitive and physical performance in physiologically demanding environments. However research into its effectiveness on cognitive and physical performance during exercise in the heat has revealed mixed findings. This study examined the effects of a commonly employed dosage of tyrosine supplementation on soccer players’ physical and decision-making performance, cognitive appraisal, and affective states, during prolonged high-intensity intermittent exercise in hot conditions. Eight trained male soccer players completed a 92-minute high-intensity intermittent cycling sprint protocol whilst responding to soccer-specific decision-making tasks at various time points in 32°C (50%rh), in two counterbalanced conditions; tyrosine (150mg.kg^-1^) and placebo. No differences were found for peak power output (*p* = .486; 715 ± 98W vs 724 ± 98W, respectively), decision-making (*p* = .627; 86.9 ± 10.7% vs 88.6 ± 7.0%, respectively), cognitive appraisal (*p* = .693, 0.90 ± 0.42 vs 0.88 ± 0.39, respectively) nor affective states (*p* = .918; 1.15 ± 1.55 vs 1.14 ± 1.70, respectively) between tyrosine and placebo conditions. Also, no condition by time interaction effects were noted for these outcomes. In sum, tyrosine supplementation was ineffective for facilitating prolonged intermittent sprint (self-paced) activity, soccer-specific decision-making, and in alleviating perceptual strain, for soccer players’ exercising in the heat. However, future research may wish to consider alternative approaches for tyrosine supplementation (e.g., timing, dosage) or induce heightened physiological strain to extend on these findings.

## Introduction

In competitive team sports including soccer, maintaining effective execution of a range of physical (e.g., sprinting) and cognitive (e.g., decision-making) skills is fundamental for successful performance [1]. Such skill execution can be impaired by several fatigue-related factors, such as thermal strain, depleted energy stores (i.e., glycogen), dehydration and neurochemical changes [2] occurring during match-play. These responses are further exacerbated when having to perform in hot environments due to heightened core temperature, increased cardiovascular strain and fluid loss often resulting in dehydration alongside amplified perceptual strain [3, 4]. Despite this, the need to train and compete in hot environments is becoming more frequent with global warming and the globalisation of sport (e.g., Tokyo 2020 Olympic Games, Qatar 2022 World Cup) [5, 6], which could further impair performance [7–10].

The Central Fatigue Hypothesis explains that prolonged exercise and/or exposure to extreme environments changes the activity of central monoamines (i.e., serotonin [5-HT], dopamine [DA], noradrenaline [NA], and adrenaline [AD]). For instance, a higher ratio of brain DA to 5-HT is suggested to be linked with increased motivation and reduced fatigue that can facilitate physical and cognitive performance [11–14]. Moreover, higher activity of the dopaminergic pathways in the hypothalamus is suggested to predict greater exercise tolerance in the heat. It has been suggested that high dopaminergic activity is thought to override the perceived effort costs (as described by Iodice et al., 2017) when experiencing physiological fatigue and may facilitate engagement in higher-effort and higher-value actions [15]. Indeed, research has shown that dopamine reuptake inhibitors can improve exercise performance, increasing heat storage and hyperthermia tolerance, potentially due to overriding inhibitory signals sent from the central nervous system which contribute to exercise cessation due to thermal strain [16]. As a result, some researchers have attempted to manipulate physical and cognitive performance through nutritional strategies to increase the dopamine-to-serotonin ratio when competing in thermally challenging environments; whereby one such nutritional strategy is through the use of tyrosine supplementation [13, 17].

Tyrosine is a non-essential amino acid found in protein-rich dietary sources which acts as a precursor to brain neurotransmitters (i.e., DA, NA, AD) [18], which play an important role in the functioning of the prefrontal cortex [19]. Oral tyrosine supplementation can increase the ratio of tyrosine to other large neutral amino acids (e.g., tryptophan), favouring its transport across the blood–brain-barrier and increasing cerebral uptake of DA and NA [18]. Therefore, tyrosine intake may help increase effort, positive affect, and reduce perceived and performance fatigue, during demanding exercise in the heat which may facilitate cognitive and physical performance. Despite the proposed mechanistic properties of tyrosine, existing research into the effectiveness of supplementation for performing in hot conditions is limited and conflicting. For instance, 150mg.kg^-1^ tyrosine supplementation consumed 1 hour before exercise was shown to improve cycling time to exhaustion by 15 ± 11% at a constant load (68 ± 5% VO_2peak_) [13], but did not improve self-paced cycling time trial performance following 60-min cycling at 57% ± 4% VO_2peak_ [20] despite similar environmental heat exposure (30˚C), exercise modality, population, and tyrosine dosage and timing. The primary difference between these studies being the fixed- vs. self-paced exercise protocols included.

The research to date suggests that tyrosine supplementation may be facilitative only when participants are experiencing heightened physiological and/or psychological strain such as during demanding prolonged exercise in warm/hot conditions. Some studies have also shown that fixed-paced exercise to exhaustion could be more psychologically stressful to engage in than self-paced exercise [21], and that catecholamine synthesis is heightened in rodents who are forced to exercise compared to those which voluntarily exercise, despite similar intensities and durations being undertaken [22]. This could help to explain why the effects of tyrosine supplementation may vary across types of protocol used. Therefore, more demanding activity could increase catecholamine synthesis and thereby increase the potential effectiveness of tyrosine supplementation on physical performance.

The effects of tyrosine on cognition in hot environments are also inconsistent. Although a review by Attipoe et al. [23] observed “favourable” effects of tyrosine on cognition in military contexts within six articles that explored its use across varying stressful conditions (e.g., cold, heat, sleep deprivation), these studies possessed several methodological limitations (e.g., lack of randomisation/allocation concealment), which led the authors [23] to conclude the evidence was insufficient to confidently determine its effectiveness. Moreover, in the only previous study to investigate tyrosine supplementation in team sport players, vigilance was improved following prolonged intermittent exercise in warm (25˚C, 40%rh) conditions [13], though tyrosine had no effect on other measures of cognition (e.g., dual-task) nor exercise performance (distance covered). Research in military personnel has also revealed that tyrosine supplementation did not facilitate cognitive function or physical performance (time to completion) on a load-carriage self-paced walking in 40˚C heat [24]. Therefore, the mixed findings from rather limited research to date makes it difficult to determine the effectiveness of tyrosine on cognitive and physical performance.

It should also be noted that cognitive performance has been typically measured either during a break or on cessation of exercise in previous research, rather than during exercise. During competition, soccer players need to simultaneously maintain cognitive functioning (e.g., decision-making) and physical performance during high-intensity intermittent exercise [25], in hot environments. However, research has yet to investigate the effect of tyrosine on cognitive performance during exercise in the heat.

### Purpose of the research

Despite some mechanistic propositions, research into the effects of tyrosine supplementation on physical and cognitive performance are rather equivocal. Researchers have yet to investigate the effects of tyrosine on cognitive performance in hot conditions whilst simultaneously performing high-intensity intermittent exercise reflective of the demands of team sports. The aim of the present study was to examine the effects of tyrosine on soccer players’ decision-making and physical performance in hot conditions, set at 32˚C to replicate recorded or expected temperatures for previous and upcoming major sporting competitions (e.g., Tokyo 2021 Olympic Games, 2018 and 2022 UEFA Women’s Euros, 2024 Paris Olympics). Notwithstanding the mixed findings in previous research, it was hypothesised that tyrosine supplementation would help maintain decision-making and physical performance in the heat, compared to placebo. Furthermore, we examined the effects of tyrosine on cognitive appraisal, affective states, and catecholamine responses (i.e., plasma metadrenalines). It was expected that tyrosine supplementation would help attenuate threat appraisals (or increase challenge appraisal), reduce negative affective states, and stimulate plasma metadrenaline synthesis (namely plasma metanephrine and normetanephrine as serum metabolite indicators of epinephrine and norepinephrine, respectively) during exercise in the heat.

## Method

### Participants

Participants were eight well-trained student male soccer players and trained a minimum of twice a week (age = 19.75 ± 0.70 years; VO_2peak_ = 46.24 ± 4.59 mL·kg^-1^·min^-1^; body composition = 12.51 ± 3.68% body fat) who were recruited via email and social media advertisements between 24-10-2019 and 25-02-2020. Data collection took place between the months of October 2019 and March 2020. Participants had an average of 13.00 (± 1.51) years of experience competing in soccer and have competed at national (*n* = 1), regional (*n* = 3) and club (*n* = 4) levels. VO_2peak_ was reported where plateau criterion for VO_2max_ was not met. Results were lower than anticipated, potentially due to tests being conducted on the cycle ergometer to coincide with the exercise conducted in the main trials. It is worth noting that this could have potentially lowered VO_2peak_ results by approximately 7–18% compared to being conducted on a treadmill [26].

This article is the second part of a two-part study. Part A examined how heat exposure (32˚C) affected soccer-specific decision-making and physical work output during intermittent sprint exercise, compared to 18˚C, which are reported in an already published article [7]. The current article reports Part B of this larger project within the same sample, which aims to address different research purposes by examining the effects of tyrosine supplementation on physical and cognitive performance in only hot conditions (32˚C). An estimated sample size of nine participants for the two-part study was determined by a power calculation for Part A, which is presented in the previous article [7]. Although testing had to be terminated early and could not recommence within a suitable timeframe due to the pandemic, a minimum target sample size of 8 participants for this phase of the research (Part B) was recruited, allowing for sufficient counterbalancing across tyrosine and placebo conditions, to align with the sample size in previous research that revealed some effects for tyrosine on cognitive performance in soccer players after exercise in the heat [13].

Participants refrained from supplementation of ergogenic aids (including energy drinks) during the study and had not performed strenuous exercise in >30°C for more than three months [27]. Participants were non-smokers, had no history of cardiovascular or respiratory problems and were required to standardize their food intake for the morning of each trial, abstain from alcohol and caffeine (24 hours), and strenuous exercise (48 hours), and maintain their regular sleep pattern (72 hours) before each trial [27]. Participants completed food and sleep diaries 48-hours prior to each trial and asked to limit their tyrosine-rich and protein-rich foods 24 hours prior to each trial; a list of foods to limit was provided [28]. No noticeable differences were identified between trials for any participant, facilitated also by the provision of food vouchers for participants to standardise their food intake for these trials. For each trial, participants arrived at least two hours post-prandial and were asked to consume 2-3L of water in the 24-hours prior to testing. Following approval from Leeds Beckett University research ethics committee, all eligible participants provided written informed consent before commencing this research.

### Experimental design and procedure

A within-subjects 2 × experimental condition (tyrosine and placebo) double-blind, cross-over design was employed. In total, participants visited the laboratory on five occasions over a 28-day period. The first visit was for familiarisation. The second and third visit were for experimental trials one and two that formed Part A of a wider project, which were completed in a temperate (18°C; 50% rh) and hot (32°C; 50% rh) environment reported in Donnan et al. [7] to address a different purpose. The fourth and fifth visits were for experimental trials 3 and 4 (Part B of the wider project reported in the present paper) to examine the effects of tyrosine on physical and cognitive performance in the heat, whereby participants completed a Tyrosine and a Placebo condition in the heat (32°C; 50% rh). Simple randomisation was employed whereby two possible supplementation condition allocations (placebo → tyrosine; tyrosine → placebo) were randomly assigned and counterbalanced by a trained member of staff, independent from the research team. Each trial for Part B (reported in this article) was completed 7 days apart and commenced in the afternoon (~1-3pm) to allow for a suitable washout period. Testing times remained consistent to control for circadian variations [29, 30].

During the first visit, participants were familiarised with all procedures and protocols for the experimental trials alongside completion of an incremental exercise test on a cycle ergometer to determine $\dot{V}O_{2\max}$ [31] (See Donnan et al. [7]). Prior to the two experimental trials relevant to this paper, participants consumed either a 150mg.kg^-1^ dose of Tyrosine supplementation added to 250mL of sugar free squash in one condition, and the placebo (250mL sugar free squash only) in the control condition [13, 24]. A double-blind procedure was used, whereby a suitably qualified staff member, independent from the research team, randomly allocated and prepared, each of the drinks administered to participants. These were consumed one hour prior to the start of the exercise protocol, aligned with previous research [13, 17]. Fluid intake was standardised in the main trials (tyrosine vs placebo) to minimise confounding hydration effects [13]; informed by each respective participants’ water consumption in the heat (968.8 ± 345.5ml) from Part A of the wider project where no supplementation was administered. To incentivise effort and offer a competitive element to reflect the nature of sport, participants were informed that whoever had the highest combined average decision-making score and physical performance across trials would be awarded with £100 [28].

### Cycling Intermittent Sprint Protocol (CISP)

**Fig 1:**
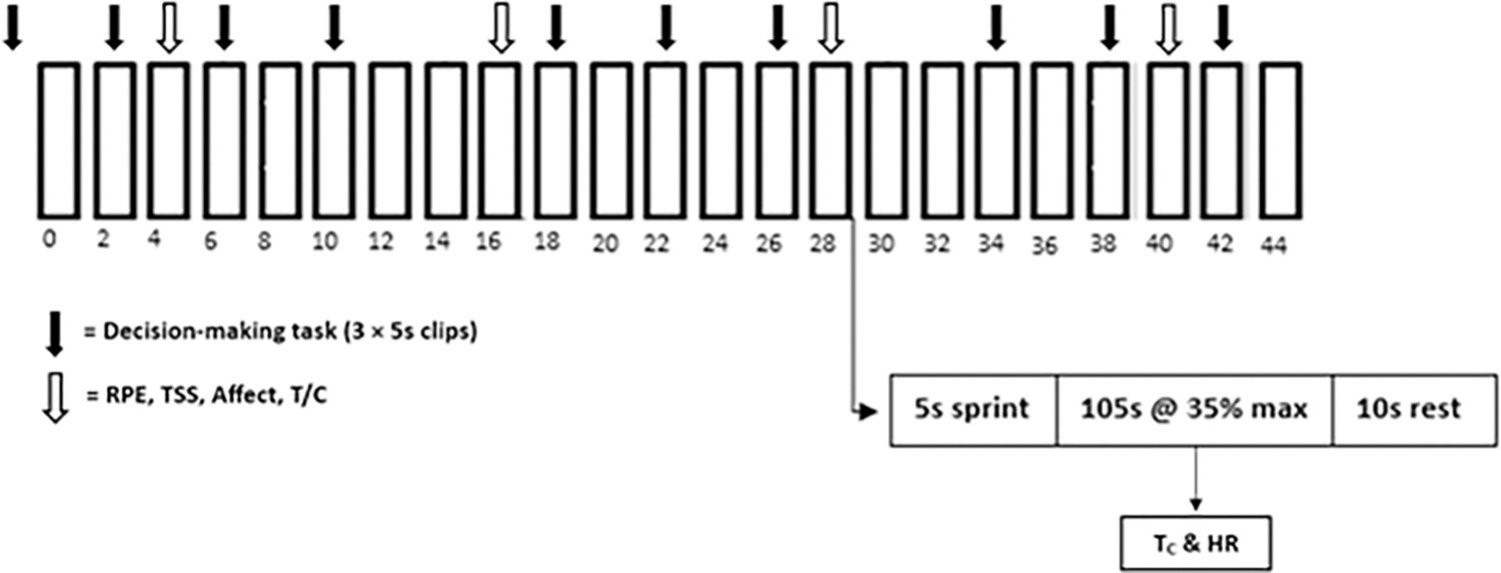
A schematic showing a modified version of one half (minutes displayed 0–44) of the extended cycling intermittent sprint protocol. Figure slightly adapted from Donnan et al. [7, p.6]. T/C = threat/challenge, T_C_ = core temperature, HR = heart rate, TSS = thermal sensation, RPE = rate of perceived exertion.

The exercise protocol used in the present research was a slightly extended version of the CISP [32] (See Fig 1). Previous research assessing the reliability of the CISP has supported its utility as a high-intensity (or sprint) intermittent exercise protocol reflective of the demands of match play in team sport players (including soccer players) and revealed strong reliability between two trails for peak power output in team sport athletes (ICC = 0.96 [0.86–0.99]) [33]. Specifically, each half of the CISP was increased to 46-minutes (23 sprints) from the original 40-minutes (20 sprints), following a standardised CISP warm-up as reported in Donnan et al. [7]. The two halves of the CISP were, interspersed by a 15-minute “half-time” period involving passive recovery in a temperate environment (~18°C).

### Physical and cognitive performance measures

Peak Power Output (PPO) in Watts (W) recorded during each sprint of the CISP [27] was used to measure physical performance. These outputs were averaged per half of the CISP for analysis.

Decision-making was measured via a previously validated soccer-specific decision-making task (see Donnan et al. [7]). Specifically, participants watched 5s video clips cut from previous World Cup footage and were asked to decide what their next action would be upon the clip freezing (e.g., “pass”, “shoot”, “clear”, and “maintain possession”). Based on expert opinion (i.e., elite coaches) from the validation process (see Donnan et al. [7]), participants were awarded 3 points for the best option, 2 points for the second best, and 1 point for any other response option given from expert opinion during the validation process (see Donnan et al. [7]). Clips were implemented at the same respective time points for each half (See Fig 1). As three clips were viewed pre-CISP and nine clips during each half of the CISP, 9 points were available pre-CISP and 27 points were available for each half of the CISP. A percentage decision making score was then calculated for pre-CISP and each of half of the CISP.

### Physiological measures

Core temperature (T_C_) was measured using a temperature sensor telemetry system (CorTemp®, HQinc, US) via a small pill ingested ~6 hours prior to exercise [34]. Heart rate (HR) (in bpm) was recorded using a polar HR monitor. Both T_C_ and HR were recorded every 2-minutes and averaged across 10-minute time points and the last 6 minutes of each half of the CISP. Nude body mass was recorded pre-post each CISP using Tanita scales (Tanita DC-430 S MA) to determine fluid loss [35]. For measurement of plasma metadrenalines, 3ml blood samples were collected in EDTA tubes from an indwelling venous catheter located in the arm pre-CISP, at half-time, and immediately post-CISP for analysis of plasma metadrenaline (MET) and normetanephrine (NMET) [7]. These samples were frozen at -80°c until liquid chromatography–tandem mass spectrometry analysis, conducted in the Integrated Laboratory Medicine Department, Freeman Hospital.

### Perceptual measures

Thermal sensation (TSS) was measured using Toner et al.’s [36] 0 (*unbearably cold*) to 8 (*unbearably hot*) Likert scale. Previous research has revealed this to be a valid measure of perceived heat stress with strong correlations (r = .72) between thermal sensation and rectal temperature [37]. Rating of perceived exertion (RPE) was recorded using the 6–20 Borg scale [38] and affective valence was measured using the Feeling Scale anchored from -5 (*very bad*) through 0 (*neutral*) to 5 (*very good*) [39]. Unick et al. (2015) found excellent agreement for RPE (ICC = 0.77) and for the Feeling Scale (ICC = 0.83) within subjects during exercise across three sessions in previous research. Perceived challenge and threat states were measured using the cognitive appraisal ratio [40]. All perceptual measures were reported at the same four time-points during each half of the CISP (see Fig 1).

### Data analysis

After data screening and checking for normality of the data using the Shapiro-Wilk test, a series of two-way (experimental condition by time) repeated measures ANOVAs were performed using IBM SPSS statistics 24.0 (IBM Corporation) for each outcome variable. Homogeneity of variance was assessed using Mauchly’s test of Sphericity, where this was unable to be assumed, Greenhouse-Geisser corrections were applied. When significant results were identified established at *p* < .05, Bonferroni pairwise comparisons post-hoc tests were performed. Partial eta-squared (η_p_^2^) and generalised eta-squared (η^2^_*G*_) are reported as the effect size for ANOVAs. Due to Cohen’s [41] original partial eta-squared benchmarks not being suitable for interpreting partial eta-squared for multi-factorial within-subjects designs [42], we employed the original benchmarks of .01, .06 and .14 for small, medium and large effect sizes, respectively, to generalized eta-squared as per Lakens [42]. Cohen’s *d* [43] was reported for any post-hoc pairwise comparisons and values of 0.2, 0.5 and 0.8 indicated small, moderate, and large effects for pairwise comparisons, respectively. As the main time effects for the same exercise protocol in the same sample were included in a previous article [7], we only focus on the main condition (tyrosine vs. placebo) and condition × time interactions in the present article. Data is reported as mean (M) ± standard deviation (SD). Supplementary figures are also available to show the individualised responses for the main outcome measures as raincloud plots (S1–S4 Figs).

## Results

### Performance measures

There were no condition, or condition × time interaction, effects for physical performance (i.e., peak power output) nor decision making (%) (Table 1) (with effects being reflective of small effect sizes or weaker than small effect sizes).

**Table 1:** Results from 2-way repeated-measures ANOVAs for each variable.

Variables	Condition								Condition × time			
	*F*(df)	*p*	η_p_^2^	η^2^_*G*_	Tyrosine		Placebo		*F*(df)	*p*	η_p_^2^	η^2^_*G*_
					*M*	*SD*	*M*	*SD*				
**Peak Power Output** (W)	_(1,7)_ = 0.540	.486	.072	.002	715	98	724	91	_(1,7)_ = 0.637	.451	.083	≤ .001
**Decision Making** (%)	_(1,7)_ = 0.259	.627	.036	.005	86.87	10.66	88.60	7.01	_(2,14)_ = 0.459	.166	.226	.055
**T**_**C**_ **(**˚C)	_(1,7)_ = 0.208	.662	.029	.014	38.03	0.25	37.96	0.20	_(9,63)_ = 1.064	.401	.132	.011
**HR** (bpm)	_(1,7)_ = 1.215	.307	.148	.006	159	12	156	8	_(9,63)_ = 1.007	.444	.126	.053
**NMET** (pmol/L)	_(1,7)_ = 0.009	.926	.***001***	≤ .001	909.79	327.76	900.42	283.61	_(2,14)_ = 1.485	.260	.175	.029
**MET** (pmol/L)	_(1,7)_ = 3.253	.114	.317	.067	251.25	68.87	295.88	88.21	_(2,14)_ = 4.065	.041*	.367	.044
**Fluid Loss (ml)**	_(1,7)_ = 0.257	.628	.035	.004	-1.52	0.34	-1.56	0.32				
**Urine Osmolality (mOsm)**	_(1,7)_ = 3.141	.120	.310	.032	0.32	0.14	0.40	0.21	_(1,7)_ = 0.000	.987	≤ .001	≤ .001
**Challenge and Threat Ratio**	_(1,7)_ = 0.169	.693	.024	≤ .001	0.90	0.42	0.88	0.39	_(8,56)_ = 0.705	.686	.091	.007
**Affective Valence** (-5 to 5)	_(1,7)_ = 0.011	.918	.002	≤ .001	1.15	1.55	1.14	1.70	_(8,56)_ = 0.132	.998	.019	≤ .001
**RPE** (6 to 20)	_(1,7)_ = 0.346	.575	.047	.002	13.41	1.24	13.27	1.67	_(7,49)_ = 0.745	.635	.096	.009
**TSS** (0 to 8)	_(1,7)_ = 0.173	.690	.024	.005	5.92	0.51	5.84	0.45	_(4,79)_ = 0.687	.682	.089	.011

*Note*: T_C =_ core temperature, HR = heart rate, NMET = normetanephrine, MET = metanephrine, RPE = rate of perceived exertion, TSS = thermal sensation, * = *p* < .05, η_p_^2^ = partial eta squared, η^2^_*G*_ = generalised eta squared. For challenge and threat state ratio, a value greater than 1 indicates a threat state and a value under 1 represents more of a challenge state.

### Physiological measures

There were no condition, or condition × time interaction, effects for T_C_, HR, urine osmolality, fluid loss, or NMET (Table 1), with all effects being reflective of up to (or weaker than) small effect sizes. No main effect for condition was observed for the MET results, but a condition × time interaction was identified with a medium effect size (Table 1). Specifically, MET concentration was higher at half-time in the placebo compared to tyrosine (*M difference* = +94.25 ± 72.75pmol/L, *p* = .008, *d* = 1.12, 95% CI 33.43 to 155.07), but was not different between conditions pre-CISP (*M difference* = +10.75 ± 70.14pmol/L, *p* = .678, *d* = 0.14, 95% CI -47.89 to 69.39) or post-CISP (*M difference* = +28.88 ± 109.88pmol/L, *p* = .482, *d* = 0.27, 95% CI -62.99 to 120.74).

### Perceptual measures

There were no condition, or condition × time interaction, effects for challenge and threat state, affective valence, RPE or TSS (Table 1). All effects were reflective of up to (or weaker than) a small effect size.

## Discussion

Although tyrosine has been proposed to potentially have some ergogenic effects on physical and cognitive performance in physically demanding environments, research has yet to examine the effects of tyrosine supplementation on cognitive performance during exercise in athletes. The present study investigated the effect of a commonly employed dosage of tyrosine supplementation [13] on physical and cognitive performance in soccer players during high-intensity intermittent exercise, reflective of the physical effort demands of team sport [32], in the heat. In sum, 150mg.kg^-1^ tyrosine consumed 1 hour prior to exercise, elicited no physical (peak power output) or cognitive (decision-making) benefits during prolonged intermittent sprint exercise in the heat. There were also no differences in perceptual measures observed between conditions (affect, TSS, RPE, demand/resource appraisal). Further, there were no significant physiological differences between conditions, other than plasma MET concentration being lower at half-time with Tyrosine compared to placebo.

No effects on physical performance were found following tyrosine supplementation compared to a placebo. These findings align with most of the limited existing research exploring the use of tyrosine on physical performance indices [13, 20, 23, 24, 44], though contradictory results have been observed [17]. For instance, Tumilty et al. [17] observed that 150mg•kg•bm^-1^ of tyrosine improved cycling time to exhaustion by ∼15% in 30˚C. However, Tumilty et al. [20] later found no differences between tyrosine and placebo conditions for a self-paced cycling time-trial performance in 30˚C. It is possible that fixed-paced exercise could be more psychologically (and physiologically) stressful than self-paced exercise [21], which could partly explain these discrepancies, or could be dependent on the nature of the physical performance (such as the intensity of exertion). Future research investigating the effects of tyrosine on physical performance measures based on differing levels of physiological demands of exercise in the heat could offer deeper insights into these discrepancies.

Similarly, we found no effects on the physical indices of T_C_ and HR during exercise in the present research. However, we did identify significantly lower metanephrine concentration in hot conditions with tyrosine compared to placebo at half-time, indicating reduced sympatho-adrenal medullary activation with tyrosine compared to placebo [45]. Tyrosine concentration has been shown to peak two hours after single-dose (150mg•kg•bodymass-1) supplementation [46, 47]. In this study, participants consumed tyrosine one hour prior to exercise, to try to facilitate performance immediately after half-time, where previous cognitive and physical decrements had been observed [48]. This may offer some explanation as to why metanephrine concentration was lower at half-time with tyrosine, but not post-CISP. Heightened epinephrine and norepinephrine levels have previously been associated with additional stress reactivity and reduced perceived control and self-efficacy [49] and these factors have also been shown to provoke more of a threat state. That said, we found no effects of tyrosine on cognitive appraisal (challenge/threat state), affective valence nor other perceptual measures in the present study (e.g., perceived exertion and thermal straight).

In the only other previous study investigating the use of tyrosine on cognitive function (albeit during a break or following exercise) using a prolonged intermittent sprint protocol in soccer players, tyrosine supplementation improved vigilance in comparison to placebo, though tyrosine failed to have an effect on more higher order cognitive functions (e.g., dual-task) [13]. Despite a lower environmental temperature used (~25˚C) than the present study, Coull et al. [13] implemented a fixed-pace, prolonged intermittent running protocol where participants experienced greater levels of physiological strain (i.e., *peak* T_C_ = ~39˚C vs ~38.4˚C), likely due to running activity inducing additional metabolic heat production compared with cycling-based activity [50]. Therefore, it is possible tyrosine may be more likely to have cognitive benefits (alongside potentially having effects on appraisal and affective valence) during exercise when greater levels of physiological and psychological strain are induced (e.g., via fixed paced and running based protocols), thereby increasing the need for additional tyrosine stores.

### Limitations and future research

A cycling intermittent sprint protocol was used to allow players to modulate their own high-intensity physical work output as per match-play, whilst simultaneously investigating cognitive performance. Despite this being widely employed within previous team sport literature [32, 33], the physiological stress response to the heat could have been more noticeable during running-based activity [51]. This would better replicate the demands of match-play and potentially increase the need for additional tyrosine stores. Also, although we attempted to incentive effort during the exercise protocol employed in the present study by inducing a competitive element and a financial incentive [52], this does not truly replicate the competitive nature of team sport beyond the laboratory [53]. Further, there was no formal assessment of the effectiveness of the blinding of participants in the current research, and despite using a similar placebo to that implemented in existing research, we cannot be certain whether placebo effects may have contributed to our findings [54]. Therefore, although the tyrosine dosage examined in the present study did not affect peak power output, decision making, cognitive appraisal and affective valence, these factors should be considered when interpreting these findings. Future research could investigate the use of tyrosine supplementation (potentially employing different dosages) on soccer players’ physical and decision-making performance in the heat using an unfixed, intermittent running protocol to potentially better replicate the physiological and psychological strain experienced during match-play or explore its use beyond the laboratory in competitive contexts.

## Conclusion

This research was the first to examine the effects of a commonly employed dosage of tyrosine on decision making, cognitive appraisal and affective valence during exercise in the heat. We examined these effects in soccer players whilst engaging in high-intensity intermittent exercise protocol reflective of the physical demands of team sport [27], and found no effects of tyrosine on power output, decision making, cognitive appraisal nor affective valence. Although metadrenaline concentration levels were lower at half-time following tyrosine consumption, these were not found post-exercise. It is possible that the null effects found in this study for acute tyrosine supplementation could be due to the demands of the exercise protocol employed being insufficient to demonstrate the potential ergogenic benefits of tyrosine. Given the limited research in this area, further investigation is warranted to examine the effects of tyrosine on physical and cognitive performance during exercise especially employing more strenuous physical demands and heat strain (e.g., where T_C_ reaches ~39˚C). Further examination into the dosage and timing of dosage intake would also be beneficial, such as exploring larger (and safe) doses than those previously administered in the literature (300 mg.kg^-1^) which has observed cognitive benefits in warm and cold conditions (respectively) where no adverse tyrosine-related supplementation side effects are noted [13, 55].

## Supporting information

S1 Fig Individual responses for mean decision-making accuracy (%) across tyrosine and placebo conditions. (PNG)

S2 Fig Individual responses for mean cognitive appraisal (demand/resource) ratio [40] across tyrosine and placebo conditions. (PNG)

S3 Fig Individual responses for mean peak power output (W) across tyrosine compared to placebo conditions. (PNG)

S4 Fig Individual responses for mean core temperature across tyrosine compared to placebo conditions. (PNG)
