# Supplementary figures and images for: Tyrosine supplementation is ineffective in facilitating soccer players’ physical and cognitive performance during high-intensity intermittent exercise in hot conditions
Source: PLoS One. 2025 Jan 16;20(1):e0317486. doi: 10.1371/journal.pone.0317486 (PMC11737745; doi:10.1371/journal.pone.0317486)

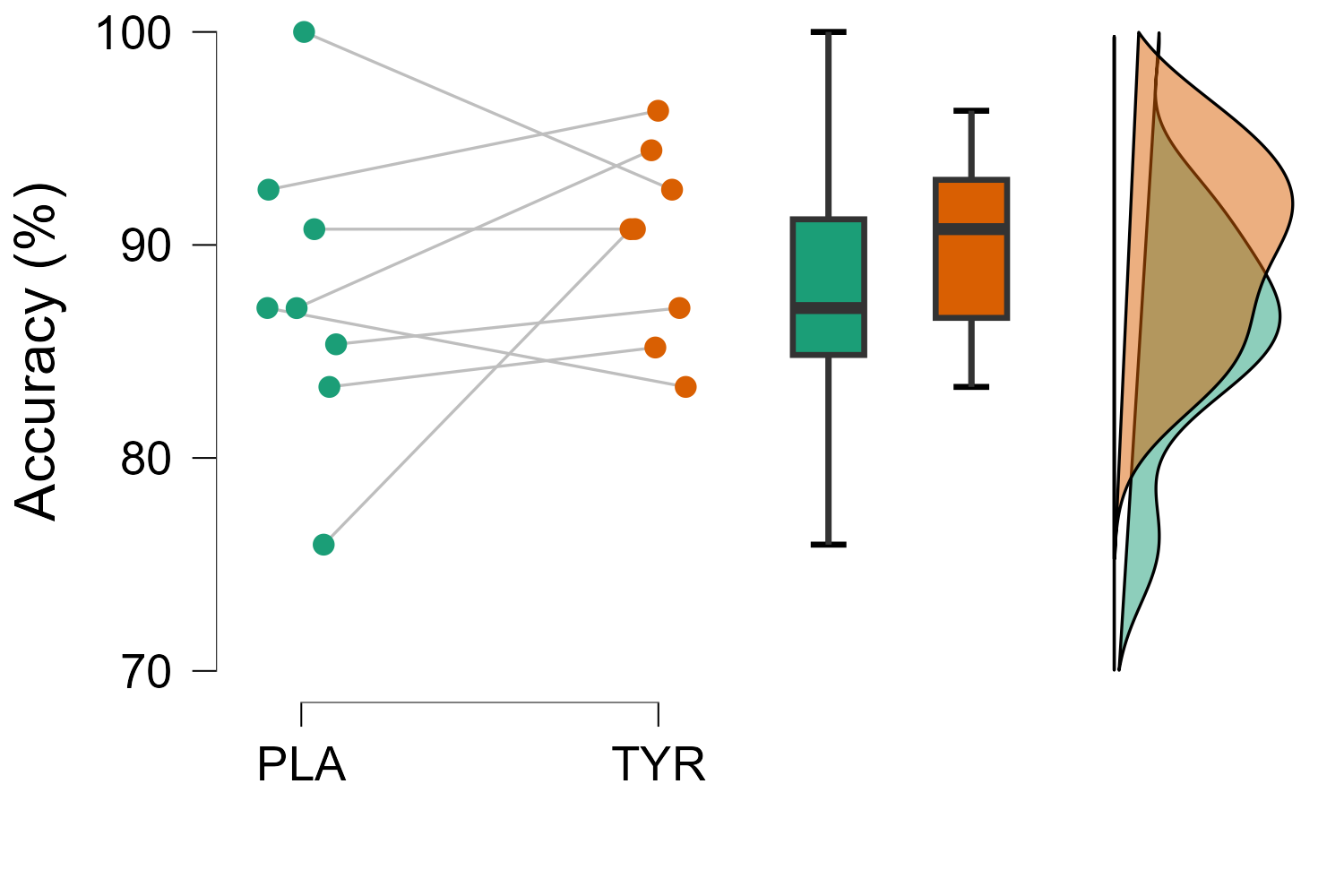

Supplement: S1 Fig — (PNG) [file pone.0317486.s001.png]

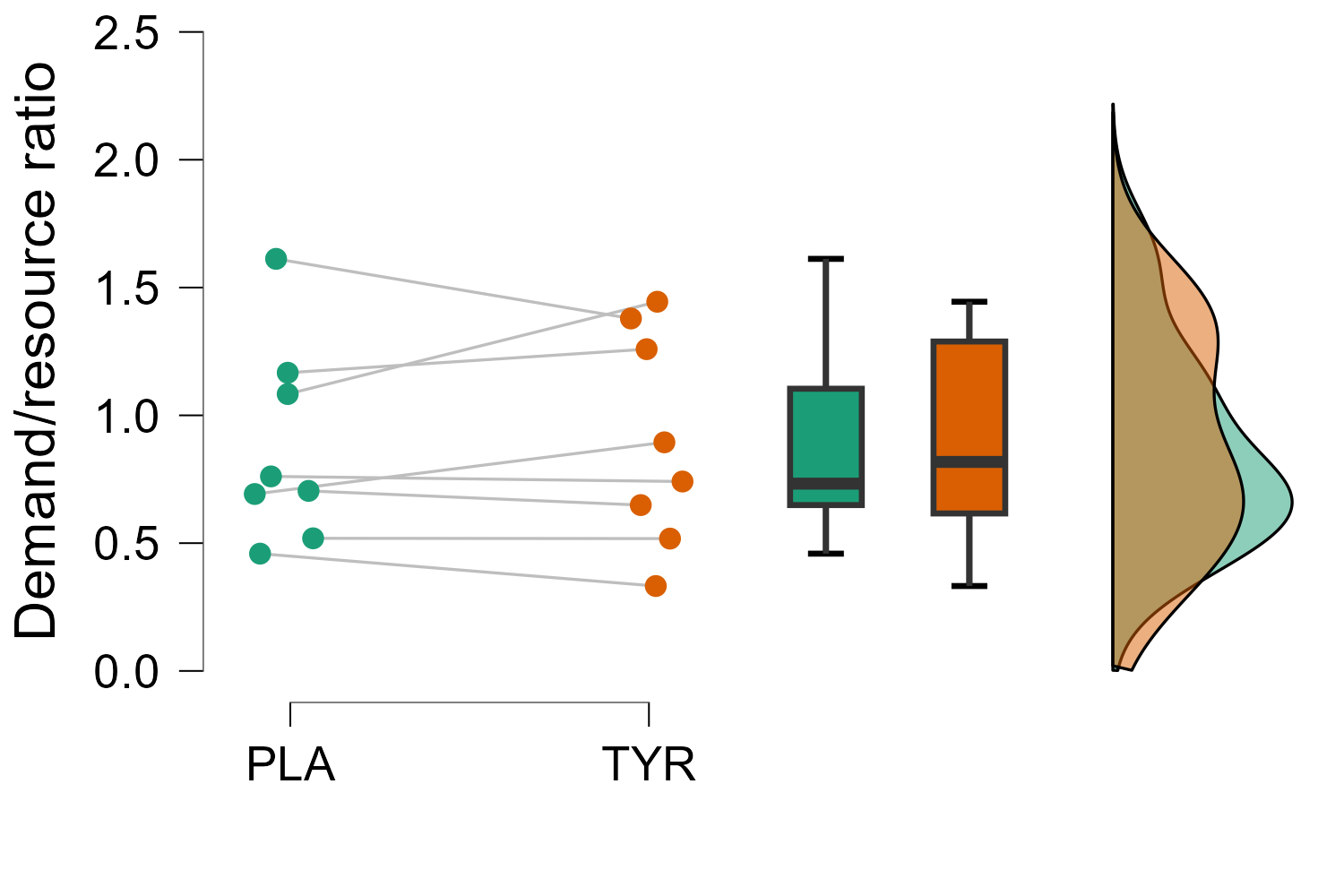

Supplement: S2 Fig — (PNG) [file pone.0317486.s002.png]

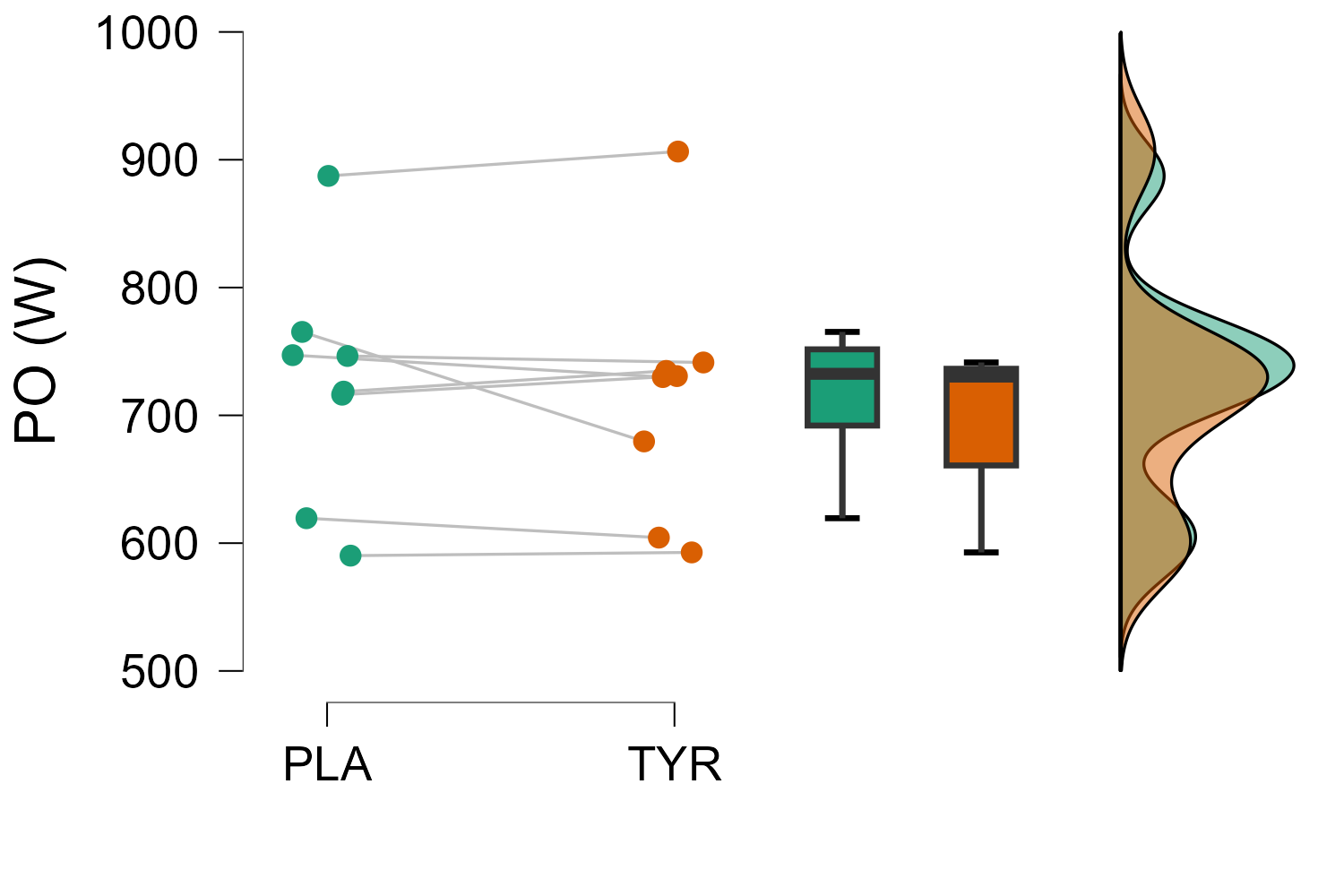

Supplement: S3 Fig — (PNG) [file pone.0317486.s003.png]

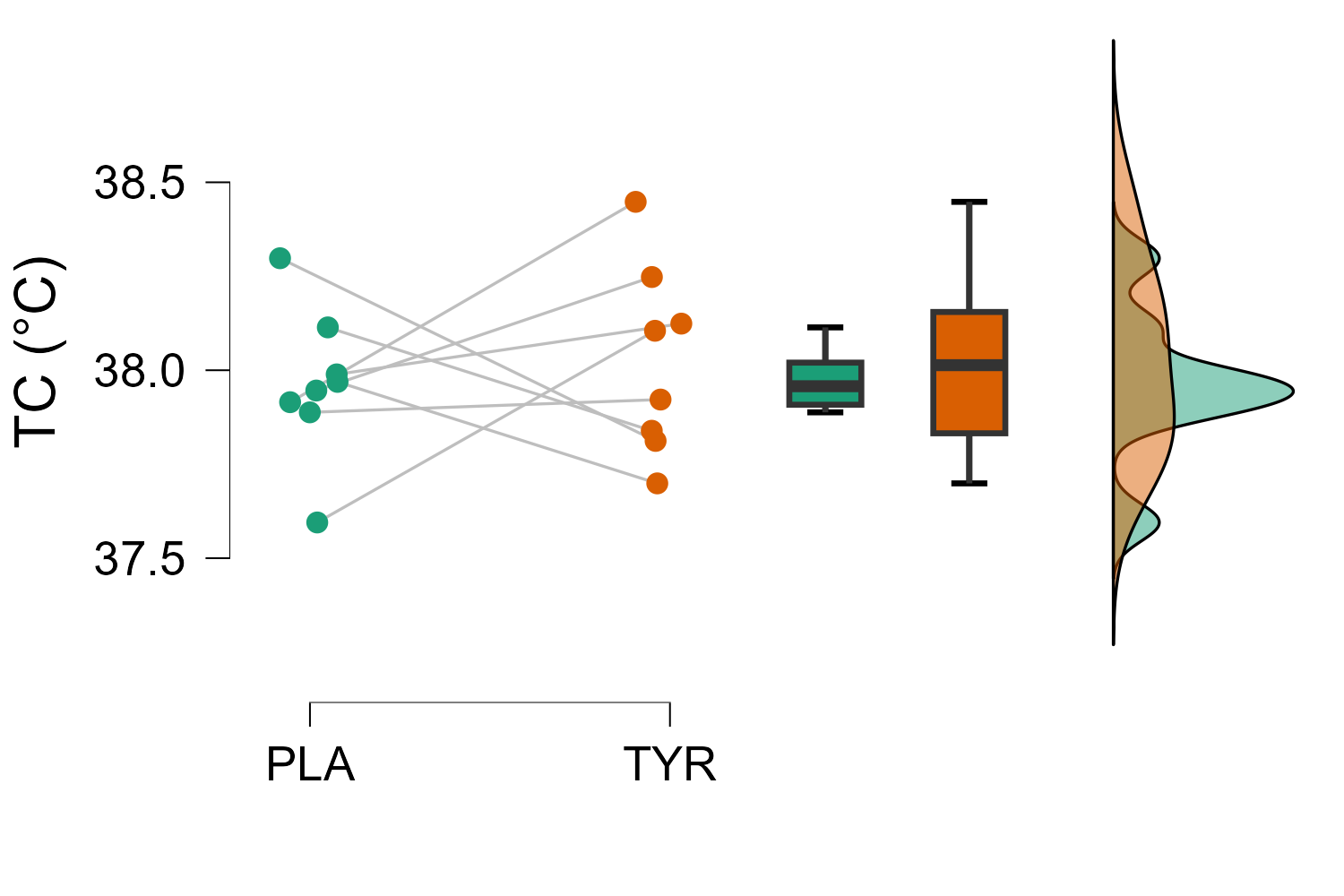

Supplement: S4 Fig — (PNG) [file pone.0317486.s004.png]
